# Are Trade-Offs Among Species’ Ecological Interactions Scale Dependent? A Test Using Pitcher-Plant Inquiline Species

**DOI:** 10.1371/journal.pone.0041809

**Published:** 2012-07-23

**Authors:** Jamie M. Kneitel

**Affiliations:** Department of Biological Sciences, California State University, Sacramento, Sacramento, California, United States of America; University of San Diego, United States of America

## Abstract

Trade-offs among species’ ecological interactions is a pervasive explanation for species coexistence. The traits associated with trade-offs are typically measured to mechanistically explain species coexistence at a single spatial scale. However, species potentially interact at multiple scales and this may be reflected in the traits among coexisting species. I quantified species’ ecological traits associated with the trade-offs expected at both local (competitive ability and predator tolerance) and regional (competitive ability and colonization rate) community scales. The most common species (four protozoa and a rotifer) from the middle trophic level of a pitcher plant (*Sarracenia purpurea*) inquiline community were used to link species traits to previously observed patterns of species diversity and abundance. Traits associated with trade-offs (competitive ability, predator tolerance, and colonization rate) and other ecological traits (size, growth rate, and carrying capacity) were measured for each of the focal species. Traits were correlated with one another with a negative relationship indicative of a trade-off. Protozoan and rotifer species exhibited a negative relationship between competitive ability and predator tolerance, indicative of coexistence at the local community scale. There was no relationship between competitive ability and colonization rate. Size, growth rate, and carrying capacity were correlated with each other and the trade-off traits: Size was related to both competitive ability and predator tolerance, but growth rate and carrying capacity were correlated with predator tolerance. When partial correlations were conducted controlling for size, growth rate and carrying capacity, the trade-offs largely disappeared. These results imply that body size is the trait that provides the basis for ecological interactions and trade-offs. Altogether, this study showed that the examination of species’ traits in the context of coexistence at different scales can contribute to our understanding of the mechanisms underlying community structure.

## Introduction

Niche differences, exhibited as trade-offs among species, are one of the most common explanations for species coexistence in communities [1]–[7]. Trade-offs are exhibited as differences in traits that result in a negative correlation or inverse ranking among species [1], [4], [8]. For example, a trait (e.g., competitive ability) of species within a guild comes at the cost of investment in another trait (e.g., predator tolerance). Numerous traits have been identified as potential axes along which trade-offs occur, and these niche differences can ultimately lead to species coexistence at different spatial scales [4].

Species within a community potentially interact at local (within community) and regional (among local communities) spatial scales [9]–[13]. The nature of predicted trade-offs will differ according to the scale over which species interact with each other and their environment [4]. At the local community scale, numerous models predict differences in species’ resource use [14], but within a food web, the most commonly considered trade-off is between competitive ability and predator tolerance [3], [15]. When all local communities are homogenous at the regional scale, a trade-off between competitive ability and dispersal ability is required for species coexistence [16]. When local communities are heterogeneous in a region, several situations can lead to coexistence [17], including specialization on different habitat types (i.e. species sorting model) [12]. Additionally, interactions at one scale can influence interactions at another scale; for example, predators in local communities can relax the competition-colonization trade-off to facilitate coexistence at the regional scale [9]. While trade-offs alone do not guarantee coexistence, there is the potential for scale-dependent trade-offs whereby traits reflect species coexistence at local or regional scales [9], [18], [19]. Few examples exist of trade-offs at multiple scales, but an empirical examination of species traits within this framework may broaden our understanding of community structure and diversity patterns [20], [21].

Species trade-offs have been linked to relative abundance patterns in communities [22]. Likewise, the diversity patterns at different scales (alpha, beta, and gamma diversity) may reflect the pattern of coexistence resulting from trade-offs [4]. For example, trade-offs that lead to coexistence at the local scale may result in a pattern of high alpha diversity, but low beta diversity, whereas coexistence at the regional scale may produce low alpha diversity, but high beta diversity. Consequently, the interpretation of trade-offs should be assessed within the context of natural diversity patterns.

The species that inhabit the leaves of the pitcher plant, *Sarracenia purpurea*, are ideal for studying communities at different spatial scales [11], [23]–[26]. Bacterivores, protozoa and rotifers, share the same resources (bacteria) and predator (larvae of the pitcher-plant mosquito, *Wyeomyia smithii*). These species have been found to respond differently to ecological interactions at the local (competition and predation) and regional (dispersal) scales and consequently species diversity also varies according to this environmental variation [11], [23], [27]–[29]. Within-leaf (local community) diversity tends to be low, but high species turnover among plants (beta diversity) has been commonly observed [24], [25], [27]. Community structure (resource levels and predator densities) is also spatially variable [30]–[32], which potentially provides the environmental backdrop for these observed diversity patterns [24], [25], [27]. The question, however, still remains whether species in this community differ in their ecological traits, thereby providing a mechanism for the observed abundance and diversity patterns.

I examined several trade-offs predicted to lead to coexistence at local and regional scales to understand the potential mechanisms underlying the pattern of species diversity. I tested the hypothesis that species will exhibit trade-offs indicative of coexistence at both the local and regional spatial scales by measuring species traits (competitive ability, predator tolerance, and colonization rate) for the 5 most common bacterivore species of the pitcher-plant community. Competitive ability and predator tolerance were used as traits indicative of coexistence at the local community scale, while competitive ability and colonization rates were used as traits indicative of coexistence at the regional scale. Traits that were negatively correlated would be evidence for trade-offs; this would provide one possible explanation for the patterns of species abundance and diversity previously observed in this system [11], [23], [27]. The linking of body size, per capita growth, and carrying capacity to the interaction traits (competitive ability, predator tolerance, and colonization rate) has been a ubiquitous question in ecology [33]–[37] and were also measured to determine relationships among these traits.

## Methods

The inquiline community that occurs inside the leaves of the pitcher plant *Sarracenia purpurea* has been well described in other studies [23], [27], [28], [30]–[32], [38]–[40] and will be described only briefly here. The pitcher leaves collect rainfall and then act as pitfall traps for insects and other invertebrates, which are the primary energy input to the system. Drowned prey are decomposed by bacteria, which feed the higher trophic levels. The species of the middle trophic level (protozoa and rotifers) share a common resource (bacteria) and predator (larvae of the pitcher plant mosquito, *Wyeomyia smithii*). The five most common (*sensu* abundance and distribution) species of the middle trophic level are the protozoa *Bodo* sp., *Poterioochromonas* sp., *Colpidium* sp., *Colpoda* sp., and the pitcher-plant obligate rotifer, *Habrotrocha rosa*.

No permits were required for the described field studies below. Further, specific permissions were not required for these locations because they were on public lands and not protected. None of the field or laboratory studies involved endangered or protected species.

### Colonization Rates

Colonization rate measurements were conducted on 42 randomly selected pitcher leaves (on different plants) at the Crystal Site in the Apalachicola National Forest in northern Florida. It is very difficult to determine the source of protozoan and rotifer colonists in pitcher plants, but their colonization into the communities are likely similar to other aquatic communities (e.g., ponds) [41], [42]. I cleaned 31 of the leaves by removing contents, rinsing repeatedly with sterile water, washing with a 30% hydrogen peroxide solution, and rinsing again repeatedly with sterile water. Each leaf was then filled with ∼15 ml of sterile water. The other 11 leaves were newly opened and hence did not receive a cleaning treatment. There was no significant difference in colonization rates between cleaned and newly opened leaves except for *Colpoda* (*F* = 3.25, *P* = 0.012), which had higher rates in cleaned leaves; care should thus be taken when interpreting *Colpoda* colonization results.

I sampled daily for 4 days and then every other day for a further 10 days. Leaf contents were gently stirred, a 1-ml sample was taken, and the same volume of sterile water added. In the laboratory, I identified the species present in each pitcher leaf and recorded the number of leaves occupied by each focal species. Each species exhibited a saturating curve quickly (<7 days), and therefore only data for days 1 through 7 were used. While population growth rates of each species may bias these measurements, this was unlikely because colonization and growth rates were not correlated. Nonetheless, I could not exclude the influence of population growth on colonization rate. Colonization rate for each species was determined by first taking the slope (constrained through zero) of the number of pitcher plants colonized through time. At the site scale, species’ percent occupancy and average abundance of species at the site were not correlated to species’ colonization abilities (n = 37; percent occupancy: r = −0.81, *P* = 0.10; abundance: r = −0.47, *P* = 0.43). Nevertheless, colonization rates of each species were divided by their percent occupancy; this adjustment relieves the rate from any bias resulting from high regional abundance.

**Figure 1:**
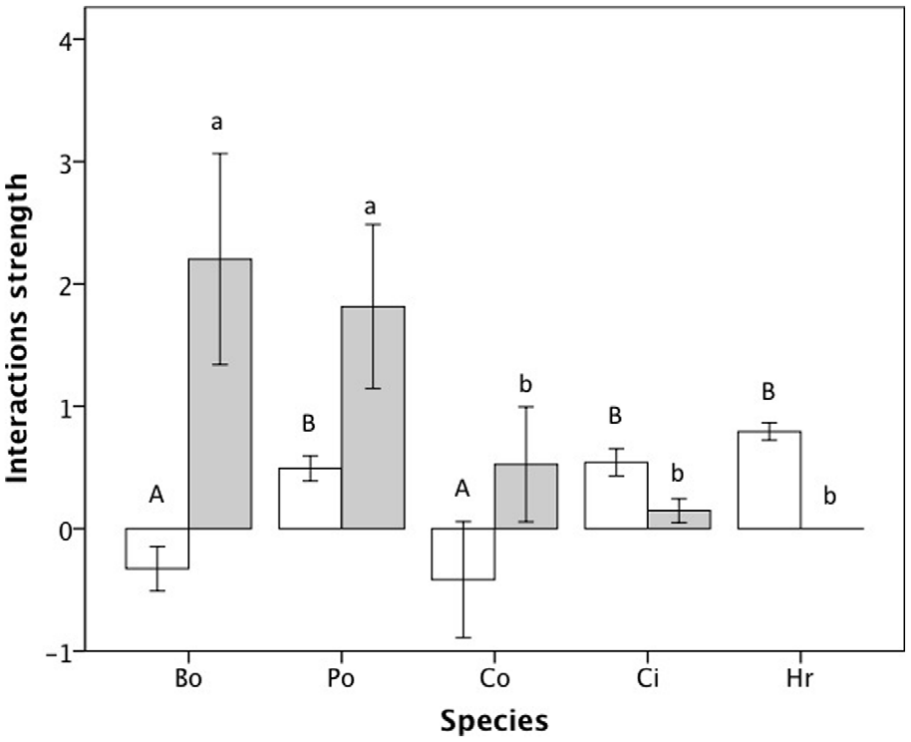
Mean interactions strength (± SE) of each species’ competitive ability (white bars) and predator tolerance (grey bars). Higher values represent increased strength of that ecological trait. Species sharing a letter were not significantly different from each other based on Bonferroni post hoc tests (*P* = 0.05). The upper case letters are associated with competitive ability and the lower case letters are associated with predator tolerance. Species abbreviations are: Bo, *Bodo* sp.; Po, *Poterioochromonas* sp.; Ci, *Colpidium* sp.; Co, *Colpoda* sp.; Hr, *Habrotrocha rosa*.

### Establishment of Monocultures

A few individuals of each focal species (*Bodo* sp., *Poterioochromonas* sp., *Colpidium* sp., *Colpoda* sp., and *Habrotrocha rosa*) were isolated from field samples and used to establish monocultures. Monocultures were kept in 50-ml vials, each containing 30 ml of sterile water and ∼20 dead sterilized ants, and were maintained by weekly serial transfers to new vials. These cultures were set up to provide stock populations for the competition and predation experiments. Except for *H. rosa*, the individuals could be identified only to genus. The individuals were morphologically indistinguishable, but it was still possible that cryptic species existed in the monocultures [43]. Nonetheless, the analyses in this study should be considered conservative because the presence of multiple species would likely result in greater trait variation and therefore a decreased chance of detecting a trade-off.

### Competition Experiment

Single-species cultures and all possible pairwise-competition cultures were established in an additive design in 50-ml vials holding 15 ml of sterile water, with four replicates of each treatment. Approximately 500 individuals of each species were used to inoculate all treatments. Five ants were added to cultures, which reflect low resource levels [11], [23]. Since rotifer growth rates are much slower than protozoa, their cultures were initiated 24 hours in advance. Samples were taken every 6 hours for 48 hours and then every 24 hours for the following 7 days. Vials were gently mixed and 0.1 ml was placed in a Palmer counting cell. Density measurements were taken under a phase-contrast microscope at 100x magnification. Mean individual size (cell or body length, in µm; n = 5) was measured in a single-species culture on day 7.

Regression analyses were conducted using population density as the independent variable and per capita growth rate as the dependent variable. I used the x-intercept as the estimate for carrying capacity and the y-intercept as the estimate for per capita growth rate (r_max_).

Population sizes at equilibrium for each species in isolation and in competition were used to calculate each species’ competitive ability. This was measured as the amount by which each species *i* was affected by its competitor *j* and was calculated as:

Competitive ability  =  (N*_i_*−N*_ij_*)/N*_i_*

where N*_i_* is the population size of species *i* grown in monoculture and N*_ij_* is the population size of species *i* when grown in competition with species *j*. A competitive interaction matrix was produced from the set of pairwise interactions. The principle diagonal was ignored, and the mean of each column was each species’ competitive ability estimate.

**Figure 2:**
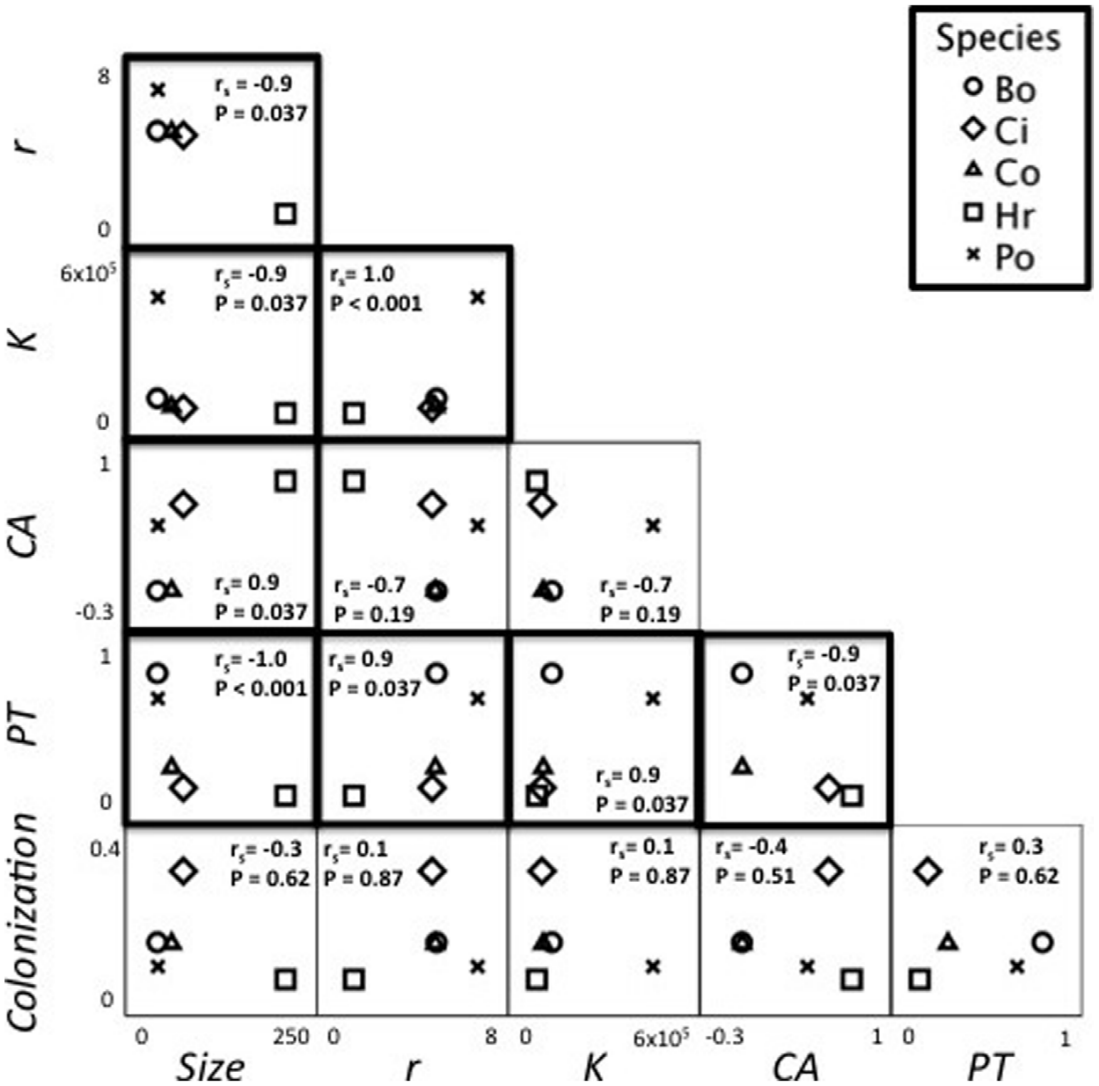
Scatterplots of measured species’ traits (Competitive Ability [CA], Predator Tolerance [PT], Colonization Rate [Colonization], Size, Growth rate [r], and Carrying capacity [K]). Bold borders indicate a significant relationship (*P*<0.05) from Spearman rank correlation, see Results for details. Correlations with competitive ability and predator tolerance address the local scale, and those including colonization address the regional scale. Species abbreviations are: Bo, *Bodo* sp.; Po, *Poterioochromonas* sp.; Ci, *Colpidium* sp.; Co, *Colpoda* sp.; Hr, *Habrotrocha rosa*.

### Predator-Tolerance Experiment

All predation experiments were conducted in 15 ml of sterile water in 50-ml vials. Each species was grown alone and with 3 *Wyeomyia smithii* larvae at low resource levels (five ants) and replicated four times. Predator densities were in the lower range of those found naturally because higher predator densities resulted in extinction for all species (J.M. Kneitel, *unpublished data*).

On day 9, the vials were sampled as described in the competition experiment above. Predator tolerance was calculated as:

Predator tolerance = N*_ip_*/N*_i_*

where N*_ip_* is the population size of species *i* when grown with the predator, and N*_i_* is the mean population size of species *i* grown in monoculture. The predator tolerance estimate then increases as the population size in the presence of a predator approaches its size in monoculture, reflecting predator tolerance.

### Statistical Analyses

To test whether interaction strengths differed among species, MANOVA was used with the dependent variables of competitive ability and predator tolerance. Data met parametric assumptions. Spearman rank correlations were conducted using the means of all species traits (competitive ability, predator tolerance, colonization rate, body size, growth rate, and carrying capacity). Since there were several correlations among species traits, I also conducted partial Spearman rank correlations among competitive ability, predator tolerance, and colonization ability while controlling for body size, per capita growth rate, and carrying capacity. Because of small sample sizes, controlled variables could be used only one at a time.

## Results

Species exhibited variation in both their competitive abilities and tolerance of predators (Figure 1). MANOVA results indicated that the species were significantly different in both these interactions (Wilks’ lambda = 0.214, *F*_(8, 28)_ = 4.06, *P* = 0.003). Both competitive abilities (*F*_(4, 20)_  = 5.3, *P* = 0.007) and predator tolerances (*F*_(4, 20)_ = 3.55, *P* = 0.031) differed among species. *Bodo* sp. and *Colpoda* sp. had significantly smaller competitive abilities compared to *Poterioochromonas* sp., *Colpidium* sp. and *H. rosa* (Figure 1). In contrast, *Bodo* sp. and *Poterioochromonas* sp. had significantly greater predator tolerance than the other species (Figure 1).

A strong negative correlation was found between predator tolerance and competitive ability (Figure 2). Species less affected by predators (i.e. *Bodo* sp. and *Poterioochromonas* sp.) were weaker competitors, while the better competitors (i.e. *H. rosa* and *Colpidium* sp.) were strongly suppressed by predators. There was no correlation between competitive ability and colonization rates (Figure 2). Body size, population growth rate, and carrying capacity were examined in relation to each other (Table 1, Figure 2). Body size was negatively correlated with population growth rate (r_s_ = −0.9, *P* = 0.037) and carrying capacity (r_s_ = −0.9, *P* = 0.037); growth rate was positively correlated with carrying capacity (r_s_ = 1.0, *P*<0.001). These traits were also correlated with the traits associated with trade-offs.

**Table 1:** Focal species measured and their mean (± SE) sizes (length), population growth rates, and carrying capacities.

Species	Size (µm)	Per capita growth rate(individuals/day)	Carrying Capacity (individuals/ml)
*Bodo*	6.00 (−)	5.43 (±0.16)	77600 (±11069)
*Poterioochromonas*	7.00 (−)	7.88 (±0.04)	606769 (±15074)
*Colpoda*	28.56 (±0.29)	5.36 (±0.03)	32914 (±1086)
*Colpidium*	35.43 (±0.69)	5.17 (±0.04)	26392 (±957)
*H. rosa*	245.25 (±2.43)	0.49 (±0.03)	22.5 (±10.3)

Body size was positively correlated with competitive ability (r_s_ = 0.9, *P* = 0.037) and negatively correlated with predator tolerance (r_s_ = −1.0, *P*<0.001). This implies that larger species were better competitors, but more negatively affected by predators. There was no relationship between colonization rate and body size (r_s_ = −0.3, *P* = 0.62). Growth rate was positively correlated with predator tolerance (r_s_ = 0.9, *P* = 0.037), but there was no relationship with competitive ability (r_s_ = −0.7, *P* = 0.19). Carrying capacity was only positively correlated with predator tolerance (r_s_ = 0.9, *P* = 0.037). Species with a higher growth rate and carrying capacity were better at tolerating predators, but there was no relationship with competitive ability.

When growth rate and carrying capacity were controlled for, there were no correlations found among the trade-off traits. When size was controlled for, competitive ability-predator tolerance correlation disappeared, but predator tolerance and colonization ability became positively correlated (*r_s_* = 0.881, *P* = 0.009).

## Discussion

Trade-offs between ecological interactions can facilitate species coexistence in communities [3], [4], [20]. There was mixed support for the predicted trade-offs among the protozoan and rotifer species of the pitcher-plant inquiline system. Species exhibited trade-offs between predator tolerance and competitive ability associated with the local-community scale. However, the trade-off indicative of coexistence at the regional community scale (competition and colonization abilities) was not detected. The ecological interactions (competition and predator tolerance) were strongly correlated with species’ traits (body size, growth rate, and carrying capacity), consistent with previous studies [36]: increased size was related to increased competitive ability, while decreased size and increased growth rate and carrying capacity were related to tolerating predators. When traits were controlled for in partial correlation, the trade-off between competitive ability and predator tolerance disappeared and a positive correlation between predator tolerance and colonization ability emerged. These results suggest that individual size, growth rate, and carrying capacity strongly influence these ecological interactions and resulting trade-offs. Further, predator tolerance consistently emerged as an important trait (Figure 2), suggesting its importance to pitcher plant inquiline community structure.

Species differences in competitive ability and predator tolerance may contribute to coexistence at the local-community scale, a condition observed in numerous taxa and communities [3], [44]–[46]. This study also provides an explanation for species abundance patterns found in previous laboratory and field studies [11], [23], [27]–[29], [38]. Species in the pitcher plant system that were less affected by predators in this study (e.g., *Bodo* sp. and *Poterioochromonas* sp.) have been dominant in communities with predators or have not responded negatively to predator presence [11], [23], [28]. Furthermore, strong competitors that were weak at tolerating predators (e.g., *H. rosa* and C*olpidium* sp.) were greatly suppressed in those communities with mosquito predators [27], [28]; the opposite was true in communities without mosquito predators [11], [23], [27], [28].

The natural patterns found in predator abundance and variation in prey-species diversity suggest that trade-offs related to coexistence may be complex. Differences in species’ abilities to compete and tolerate predators may facilitate coexistence at the local scale [3], [35], but these traits may also facilitate coexistence at the regional scale when predators and resources are heterogeneous in their densities [9], as is found in the pitcher plant community. Trade-offs at a regional scale composed of heterogeneous local communities has been a mechanism of coexistence in many systems, leading to regional segregation of species according to local-community composition (i.e. the species-sorting model) [12]. In the pitcher-plant inquiline community, pitcher leaves contain great variation in predator densities [24]–[27], and this variation combined with species’ trait variation may explain the spatial patterns of species diversity [24]–[27]: low alpha diversity and high beta diversity [11], [23], [27]. Therefore, variation in local community structure may drive patterns of species coexistence at both local and regional scales. Indeed, high among-pitcher variation has been found in both predator abundance and species beta-diversity in several field studies [11], [23], [43]. The positive relationship between predator tolerance and colonization ability, when body size is controlled for, also suggests the importance of regional scale processes for species coexistence.

This study was similar to a number of previous studies that have measured natural dispersal rates and have found that colonization differences likely do not play a role in facilitating coexistence. These studies conclude that species may perform differently among habitat types regardless of dispersal ability, lending support for segregation among heterogeneous patches [47]–[49]. These studies, along with the present study, suggest that coexistence at this regional scale under varying conditions (i.e. the species-sorting model) is a common phenomenon in communities [12].

Larger-sized species in this study had lower growth rates and carrying capacities but were better competitors than the smaller species with higher growth rates that were more tolerant of predation. The suite of traits appears to be strongly linked in this system. This relationship has been found in previous studies with both bacteria and protozoa in which small species with fast growth rates were more susceptible to interference competition [5], [36], [50], [51]. Growth rate, carrying capacity, and individual size are important species traits that can also evolve in response to competitors and predators [29], [36], [51], [52]. Recent evidence has shown that mosquito-larvae predators can select upon size and growth rate in pitcher plant protozoans [29]. This previous study measured traits within a species and found no relationship between size and growth rate, in contrast to the present study, and thus highlights differences in intraspecific and interspecific trait variation. Nonetheless, body size is well known to have a number of ecological consequences, including influencing species interactions [53].

There are several limitations of this study. Other factors can affect species diversity, such as temporal variation, disturbances, and higher-order interactions [6], [8], [54]. These could not be ruled out as important factors that lead to coexistence in this system. However, in a previous laboratory experiment, higher-order interactions did not seem to be exhibited [28]. Nonetheless, species interactions are clearly contingent on the abiotic and biotic environment in natural communities. The present study may also be biased in its limited number of focal species; the species used in this study were a subset of the total number found naturally, although they were the most common species in the community. Additionally, direct and indirect interactions within and among trophic levels can be important in this system [23] but were beyond the scope of this study. In general, a theoretical and empirical understanding of these other factors, as well as the relationship and strength of trade-offs, will illuminate the importance of species traits as it relates to patterns of diversity [2], [4], [55], [56]. These limitations aside, this study highlights the potential to understand the scale of coexistence using species traits.

Interspecific trade-offs (niche differentiation) are predicted to allow coexistence in communities. Numerous models have shown that coexistence can be scale-dependent, and different traits can be important for coexistence at different spatial scales in metacommunities [12]. Few studies have examined different trade-offs as alternative hypotheses of coexistence at different scales [20]. The results of the present study emphasize that by examining scale dependent trade-offs as alternative explanations, ecologists can better understand the possible scales of coexistence. Observed trade-offs must also be understood in the context of diversity patterns and individual traits to develop more predictive models. The scales of coexistence are expected to emerge as the patterns of alpha, beta, and gamma diversity observed in communities [4]. Linking species-specific ecological traits to community patterns at different scales is important for a mechanistic understanding of community composition, structure, and dynamics.
